# Integrative analysis of bioinformatics and machine learning to identify cuprotosis-related biomarkers and immunological characteristics in heart failure

**DOI:** 10.3389/fcvm.2024.1349363

**Published:** 2024-03-18

**Authors:** Dingyuan Tu, Qiang Xu, Yanmin Luan, Jie Sun, Xiaoli Zuo, Chaoqun Ma

**Affiliations:** ^1^ Cardiovascular Research Institute and Department of Cardiology, General Hospital of Northern Theater Command, State Key Laboratory of Frigid Zone Cardiovascular Diseases (SKLFZCD), Shenyang, Liaoning, China; ^2^ Department of Cardiology, The 961st Hospital of PLA Joint Logistic Support Force, Qiqihar, Heilongjiang, China; ^3^ Department of Cardiology, Changhai Hospital, Naval Medical University, Shanghai, China; ^4^ Department of Cardiology, Navy 905 Hospital, Naval Medical University, Shanghai, China; ^5^ Reproductive Medicine Center, Changhai Hospital, Naval Medical University, Shanghai, China; ^6^ Hospital-Acquired Infection Control Department, Yantai Ludong Hospital, Yantai, Shandong, China

**Keywords:** cuprotosis, heart failure, bioinformatics, machine learning, immune infiltration

## Abstract

**Backgrounds:**

Cuprotosis is a newly discovered programmed cell death by modulating tricarboxylic acid cycle. Emerging evidence showed that cuprotosis-related genes (CRGs) are implicated in the occurrence and progression of multiple diseases. However, the mechanism of cuprotosis in heart failure (HF) has not been investigated yet.

**Methods:**

The HF microarray datasets GSE16499, GSE26887, GSE42955, GSE57338, GSE76701, and GSE79962 were downloaded from the Gene Expression Omnibus (GEO) database to identify differentially expressed CRGs between HF patients and nonfailing donors (NFDs). Four machine learning models were used to identify key CRGs features for HF diagnosis. The expression profiles of key CRGs were further validated in a merged GEO external validation dataset and human samples through quantitative reverse-transcription polymerase chain reaction (qRT-PCR). In addition, Gene Ontology (GO) function enrichment, Kyoto Encyclopedia of Genes and Genomes (KEGG) pathway enrichment, and immune infiltration analysis were used to investigate potential biological functions of key CRGs.

**Results:**

We discovered nine differentially expressed CRGs in heart tissues from HF patients and NFDs. With the aid of four machine learning algorithms, we identified three indicators of cuprotosis (DLAT, SLC31A1, and DLST) in HF, which showed good diagnostic properties. In addition, their differential expression between HF patients and NFDs was confirmed through qRT-PCR. Moreover, the results of enrichment analyses and immune infiltration exhibited that these diagnostic markers of CRGs were strongly correlated to energy metabolism and immune activity.

**Conclusions:**

Our study discovered that cuprotosis was strongly related to the pathogenesis of HF, probably by regulating energy metabolism-associated and immune-associated signaling pathways.

## Introduction

Heart failure (HF) is the common endpoint of various cardiovascular diseases, such as coronary heart disease, cardiac rhythm disorders, congenital heart disease, valvular heart disease, cardiomyopathy, and heart infections (1). Despite great advances in understanding its molecular pathogenesis, the treatment of HF remains a significant global medical burden, leading to high rates of hospitalization and mortality. Although the etiology of HF is complex and multifactorial, there is a growing recognition of the role that chronic inflammation plays in the progression of HF (2). A study by Revelo et al. demonstrated that macrophage-mediated stimulation of angiogenesis and inhibition of fibrosis early after cardiac pressure overload can delay the progression of HF (3). In addition, the adoptive transfer of chimeric antigen receptor (CAR)-expressing T cells can result in a significant reduction in cardiac fibrosis and restoration of function after cardiac injury in mice (4). These findings emphasize the potential of immunotherapy as a promising therapeutic approach for HF.

Copper is an essential micronutrient required in many biological processes. It serves as a structural and catalytic cofactor for cuproenzymes, playing important roles in physiological functions like immunity, cell division, and protein synthesis (5). Copper dyshomeostasis, which refers to an imbalance in copper levels, has been implicated in the onset and progression of neurodegenerative diseases such as Alzheimer's disease, Parkinson's disease, and Amyotrophic lateral sclerosis (6–8). Additionally, it has been associated with several types of cancer, including triple-negative breast cancer (9), colorectal cancer (10), and lung cancer (11). The metabolism of copper is also known to be enhanced during the acute phase response in inflammatory diseases (12). Lately, an entirely new mode of copper-dependent cell death, termed cuprotosis, was described. Unlike other known cell death mechanisms, cuprotosis is characterized by copper binding directly to the lipoylated components of the tricarboxylic acid (TCA) cycle, which induces proteotoxic stress and leads to cell death ultimately (13). The TCA cycle is involved in the communication between mitochondria and the nucleus, which is critical for maintaining cardiomyocyte homeostasis. Growing evidence suggests that the disturbance in the TCA cycle is inextricably linked with cardiac dysfunction (14, 15). Some genes involved in the process of cuprotosis have been identified, providing an opportunity for the identification of crucial cuprotosis-related genes (CRGs) involved in the pathological development of HF.

Recently, the advancement of high-throughput genomic technologies, such as microarray, genome sequencing, and transcriptome sequencing, has generated enormous amounts of biological data (16). These techniques have offered new insights into the pathogenesis and potential therapeutic modality for various diseases, including HF (17). However, sequence data is characterized by high dimensionality and redundancy, making it challenging to extract meaningful information from the extensive datasets. In this context, machine learning, a powerful approach for handing complex multi-dimensional datasets, has been successfully applied to genomic data (18). Moreover, the joint analysis of different machine learning algorithms has been demonstrated to improve prediction accuracy and sensitivity over a single approach (19, 20). Currently, there are no reports on the cross-combination of machine learning in cuprotosis-related bioinformatics analysis of HF, and the role of cuprotosis in HF remains unclear.

In this study, we systematically analyzed six HF microarray datasets obtained from the Gene Expression Omnibus (GEO) database (GSE16499, GSE26887, GSE42955, GSE57338, GSE76701, and GSE79962). We identified nine cuprotosis-related differential genes and used four machine learning algorithms, namely best subset regression, regularization techniques, random forest (RF), and eXtreme Gradient Boosting (XGBoost), to develop a 3-gene diagnostic signature of CRGs. This diagnostic model could distinguish HF patients from nonfailing donors (NFDs) with an excellent degree of discrimination and calibration in both the training and validation datasets. Besides, Significant correlations were observed between CRGs and immune signature, including immune cell types and immune-related functions. Moreover, the results of enrichment analyses and immune infiltration revealed that the indicators of cuprotosis (DLAT, SLC31A1, and DLST) in HF had a strong association with energy metabolism and immune activity. These findings suggest that cuprotosis may play a crucial regulatory role in the immune microenvironment of HF.

## Materials and methods

### HF data acquisition and preprocessing

**Figure 1:**
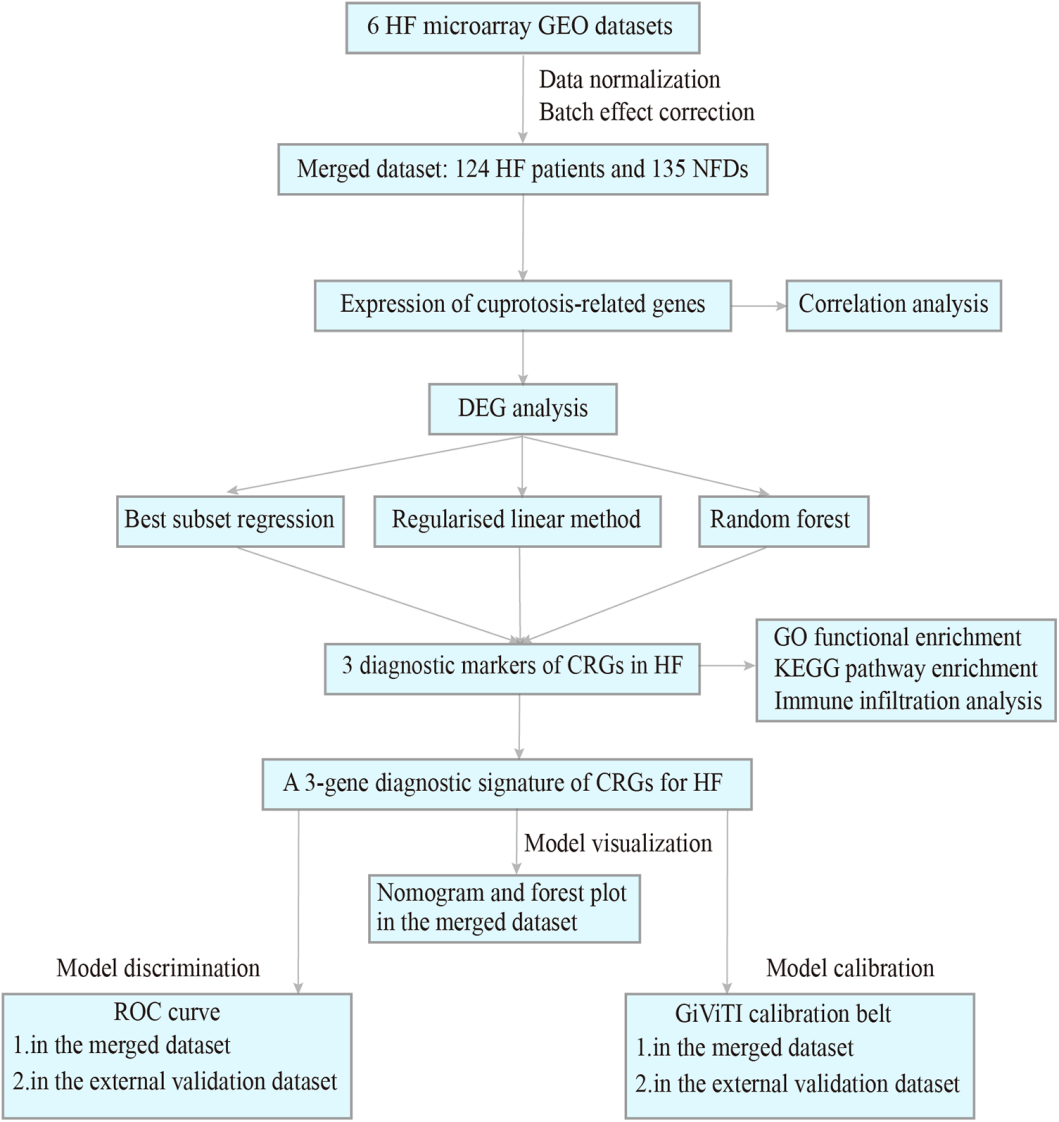
Procedure of study flowchart. HF, heart failure; GEO, gene expression omnibus; NFDs, nonfailing donors; DEG, differentially expressed gene; CRGs, cuprotosis-related genes; GO, gene ontology; KEGG, kyoto encyclopedia of genes and genomes; ROC, receiver operating characteristic.

Procedure of study flowchart is shown in Figure 1. The mRNA expression profiles of heart tissues in HF patients and NFDs were downloaded from the GEO repository (https://www.ncbi.nlm.nih.gov/geo). The following criteria were used to screen datasets: (i) the search term was set as “Heart failure”, and tissue microarrays were obtained from left ventricle of human; (ii) each dataset should contain at least four samples of HF patients and NFDs; (iii) expression information should be available within the dataset. Six datasets which met the inclusion criteria were included: GSE16499 (21) (15 HF patients, 15 NFDs), GSE26887 (22) (7 HF patients, 5 NFDs), GSE42955 (23) (24 HF patients, 5 NFDs), GSE57338 (24) (54 HF patients, 95 NFDs), GSE76701 (25) (4 HF patients, 4 NFDs), and GSE79962 (26) (20 HF patients, 11 NFDs). The raw data of these datasets were downloaded and the probe IDs were converted into gene symbols. Subsequently, the gene expression matrix was normalized and corrected by the R package “limma” (27). The six gene expression matrices were then merged, and the inter-batch effect was removed using the “sva” package (28). The batch effect, with and without adjustment, was visualized as Principal Component Analysis (PCA) plots (Supplementary Figure S1). The final merged dataset consisted of 259 samples including 124 HF patients and 135 NFDs. Additionally, GSE116250 (29) (50 HF patients, 14 NFDs), GSE71613 (30) (4 HF patients, 4 NFDs), and GSE48166 (15 HF patients, 15 NFDs) were used as independent external validation RNA-sequencing (RNA-seq) datasets. Batch effects of these three RNA-seq datasets were adjusted using the R package “RUVSeq”, which uses a generalized linear model to regress out the variation estimated from the expression of the housekeeping gene (31). Then the three RNA-seq datasets were merged as external validation dataset (Supplementary Figure S2). The characteristics of these nine datasets are listed in Supplementary Tables S1, S2.

### Identification of differentially expressed CRGs

13 CRGs were identified from previous study (13) (Supplementary Table S3). Then expression differences of CRGs between HF and NFDs samples were identified using limma. Besides, the expression relationship among differentially expressed CRGs was evaluated by Spearman correlation analysis in HF samples.

### Screen for potential diagnostic biomarkers of CRGs by machine learning

To further screen for potential biomarkers for the diagnosis of HF, machine learning was conducted on differentially expressed CRGs. Four feature selection approaches of machine learning were employed. The main steps of feature selection are described below. Firstly, HF patients and NFDs of the merged dataset were randomly assigned to a training set and an internal validation set in a 7 : 3 ratio using the R package “caret”. Ten-fold cross-validation was conducted to minimize the overfitting risk. Secondly, Subset selection in regression was applied to choose the best combination of CRGs using the sequential replacement algorithm in R package “leaps”. Then three regularised linear methods, including least absolute shrinkage and selection operator (LASSO) regression, RIDGE regression, and elastic net (EN) regression were applied to identify potential diagnostic biomarkers by the “glmnet” package, and model performance was assessed by root mean squared error (RMSE). Besides, a RF model was implemented using the “randomForest” R package. Furthermore, the R package “xgboost” was used to perform feature selection in CRGs. Eventually, potential diagnostic biomarkers were defined as the genes present in the intersection of best subset regression, RF, XGBoost and the best performing model of regularised linear methods.

### Construction and verification of a diagnostic model in HF

After the feature selection step, multivariable logistic regression models were built with the intersected CRGs, and the results were visualized by forest plot (forestplot package, R) and nomogram (rms package, R). The ability of this diagnostic model to discriminate HF patients was assessed by receiver operating characteristic (ROC) curve, and model calibration was assessed by using calibration plots. This evaluation process was conducted in the final merged dataset and external validation dataset, respectively. Additionally, the normalized expression of potential diagnostic biomarkers was extracted from the Heart Cell Atlas global heart dataset (www.heartcellatlas.org). The database is publicly available and is part of the Human Cell Atlas project, specifically focusing on Single-cell RNA sequencing (scRNA-seq) cardiac cell data.

### Validation of cuprotosis-related biomarkers in human samples using molecular biology experiments

To further validate the reliability of the cuprotosis-related biomarkers generated through bioinformatics analysis, quantitative reverse-transcription polymerase chain reaction (qRT-PCR) experiments were conducted using heart tissues and plasma samples from both HF patients and NFDs. A total of 5 ml of whole blood samples were collected from six HF patients and six NFDs into sterile sample tubes supplemented with ethylenediaminetetraacetic acid (EDTA) by experienced venipuncture nurses through the cubital vein. Following collection, the blood-containing tubes were immediately centrifuged at 1,500 g at 4°C for 10 min. After the first centrifugation step, the upper plasma phase was carefully transferred to a new tube without disturbing the intermediate buffy coat layer, which contains white blood cells and platelets. The plasma samples were then subjected to a second centrifugation step at 12,000 g and 4°C for 10 min to completely remove additional cellular nucleic acids attached to cell debris. The resulting supernatant, designated as plasma, was promptly transferred into clean polypropylene tubes after centrifugation and stored at −80°C until further use. Additionally, heart tissues from six HF patients undergoing heart transplantation were collected, along with control heart tissues from six NFDs. These heart tissues were obtained from the Specimen Bank of the Cardiovascular Surgery Laboratory and Department of Pathology at the Changhai Hospital, Shanghai, China. Written informed consent was obtained from each individual patient or their legal family members. This study was conducted in accordance with the principles of the Declaration of Helsinki and approved by the ethics committee of the Changhai Hospital. Total RNAs from heart tissues or plasma samples were isolated using Trizol reagent (Trizol™ Reagent, Invitrogen) or miRNeasy Serum/Plasma Kit (Qiagen, Cat. No. 217184), separately. Then the RNAs were reverse-transcribed into cDNAs using ReverTra Ace qPCR RT kit (TOYOBO, Japan), and cDNA was subjected to qRT-PCR quantitation using SYBR Green kit (TOYOBO, Japan). The primer sequences are listed as follows: DLAT forward, 5′-TTGAGAGCCTGGAGGAGTGT-3′ and reverse, 5′-GCCTGAGCAGAAGGTGTAGG′; SLC31A1 forward, 5′-CTGTTTTCCGGTTTGGTGAT-3′ and reverse, 5′-GGTGAGGAAAGCTCAGCATC-3′; DLST forward, 5′-GGAGATGTCAGGTGGGAGAA-3′ and reverse, 5′-GACCTTGACCACCAGGAGAA-3′. The expression levels of mRNAs relative to glyceraldehyde-3-phosphate dehydrogenase (GAPDH) or external reference were detected using the 2–*ΔΔ*Ct method.

### Correlation analysis between CRGs and immune infiltration

To evaluate the correlation between CRGs and immune activities involved in HF, single-sample gene-set enrichment analysis (ssGSEA), a method of quantifying immune infiltration levels, was adopted to analysis the merged expression data and calculate immune enrichment scores of 16 immune cell types and 13 immune-related functions. The results were displayed using correlation heatmaps.

### Enrichment analysis in diagnostic biomarkers of CRGs

In the external validation dataset of HF patients in GSE116250, potential biological functions of diagnostic biomarkers of CRGs were investigated. First, correlation analyses were conducted between diagnostic biomarkers of CRGs and other genes, and genes with the absolute value of correlation coefficient >0.5 and *p*-value <0.05 were defined as CRGs-related genes. Gene Ontology (GO) function enrichment analysis and Kyoto Encyclopedia of Genes and Genomes (KEGG) pathway enrichment analysis was conducted on these CRGs-related genes using ClusterProfiler package (32).

### Statistical analyses

For comparisons between two groups, data that were normally distributed and had equal variance were analyzed using Student's *t-*test. If the normality or variance assumptions were not met, the analysis was performed with the Wilcoxon rank sum test. Correlations were performed by Pearson correlation (normally distributed data) or Spearman correlation (non-normally distributed data). *p* < 0.05 and correlation coefficients >0.3 were considered to be meaningful correlation (33). All statistical tests were two-sided and *p* < 0.05 indicated statistical significance. R software (version 4.1.2) and its relevant packages are utilized to process, analyze and present the data.

## Results

### Datasets normalization and combination

**Figure 2:**
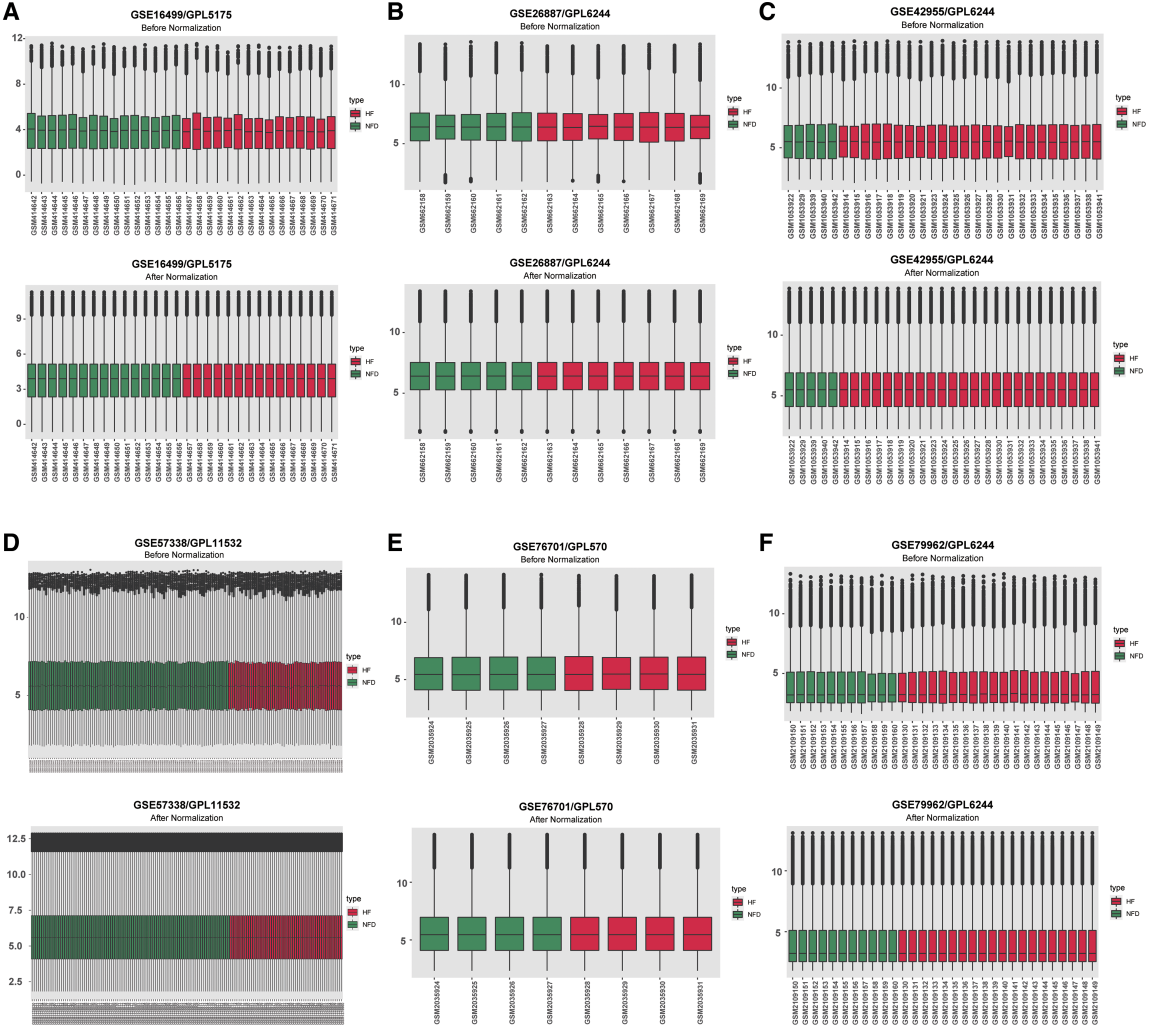
Normalizing gene expression matrices. (**A–F**) Relative mRNAs expression levels of six HF microarray datasets. Upper plots, before normalization; lower plots, after normalization. (**A**) GSE16499. (**B**) GSE26887. (**C**) GSE42955. (**D**) GSE57338. (**E**) GSE76701. (**F**) GSE79962. HF, heart failure.

The expression matrices of GSE16499, GSE26887, GSE42955, GSE57338, GSE76701, and GSE79962 were normalized, and the distribution trends of the box plots were basically straight lines (Figure 2). After datasets combination and batch effects adjustment, we ended up with a merged dataset containing 124 HF patients and 135 NFDs. Besides, the merged external validation RNA-seq dataset consisted of 69 HF patients and 33 NFDs.

### Landscape of CRGs between HF and NFDs samples

**Figure 3:**
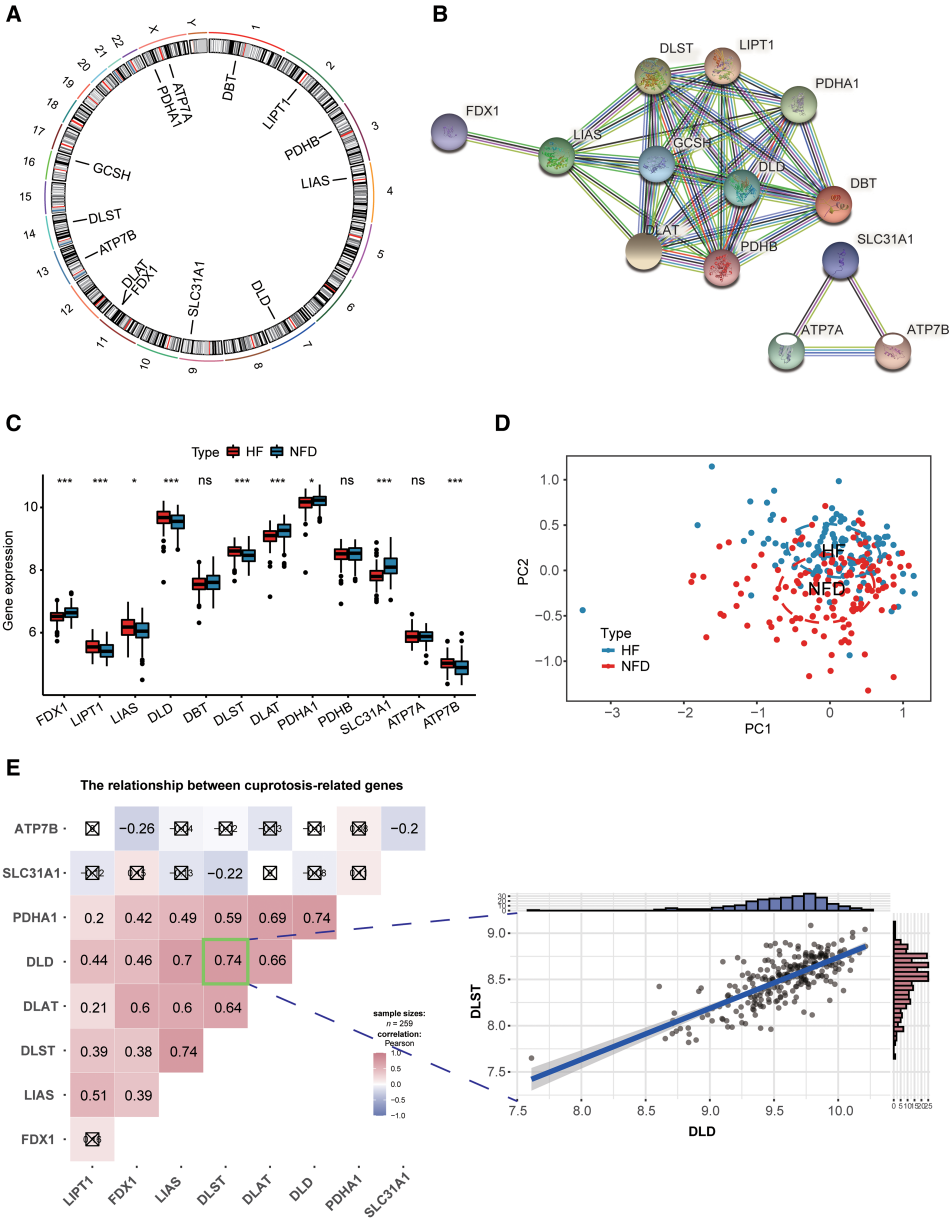
Landscape of CRGs in HF. (**A**) Circus plot showing chromosome distributions of 13 CRGs. (**B**) PPI network composed of the 13 CRGs. (**C**) The box plot demonstrating the expression profiles of CRGs between HF patients and NFDs. ns = not significant, *p < 0.05, **p < 0.01, and ***p < 0.001 vs. the NFDs group. (**D**) Principal component analysis of 9 differently expressed CRGs in HF patients from NFDs. (**E**) Correlation analysis among 9 differently expressed CRGs in HF patients. ☒ stands for non-significant at *p* < 0.05. The scatter-plot demonstrated the most correlated two CRGs: DLST and DLD. CRGs, cuprotosis-related genes; HF, heart failure; PPI, protein-protein interaction; NFDs, nonfailing donors.

Figure 3A reflected the location of 13 CRGs on chromosomes, and the protein-protein interaction (PPI) network acquired from the STRING database (https://string-db.org/) revealed a tight link among these CRGs, indicating they may function as a complex (Figure 3B). Then we observed expression patterns of 13 CRGs in heart tissues of HF patients and NFDs, and there was a significant difference in the expression of 9 CRGs. The expression levels of LIPT1, LIAS, DLD, DLST, and ATP7B were markedly higher in HF patients than NFDs, while the opposite performance of FDX1, DLAT, PDHA1, and SLC31A1was observed (Figure 3C). Figure 3D demonstrated that principal component analysis of 9 differently expressed CRGs could be used to differentiate HF patients from NFDs. The transcriptome relationships of 9 differentially expressed CRGs in HF were investigated, and we found there are close positive correlations among these CRGs. DLST-DLD was the most correlated pair, indicating that they may function together (Figure 3E).

### Screening diagnostic markers of CRGs by feature selection

**Figure 4:**
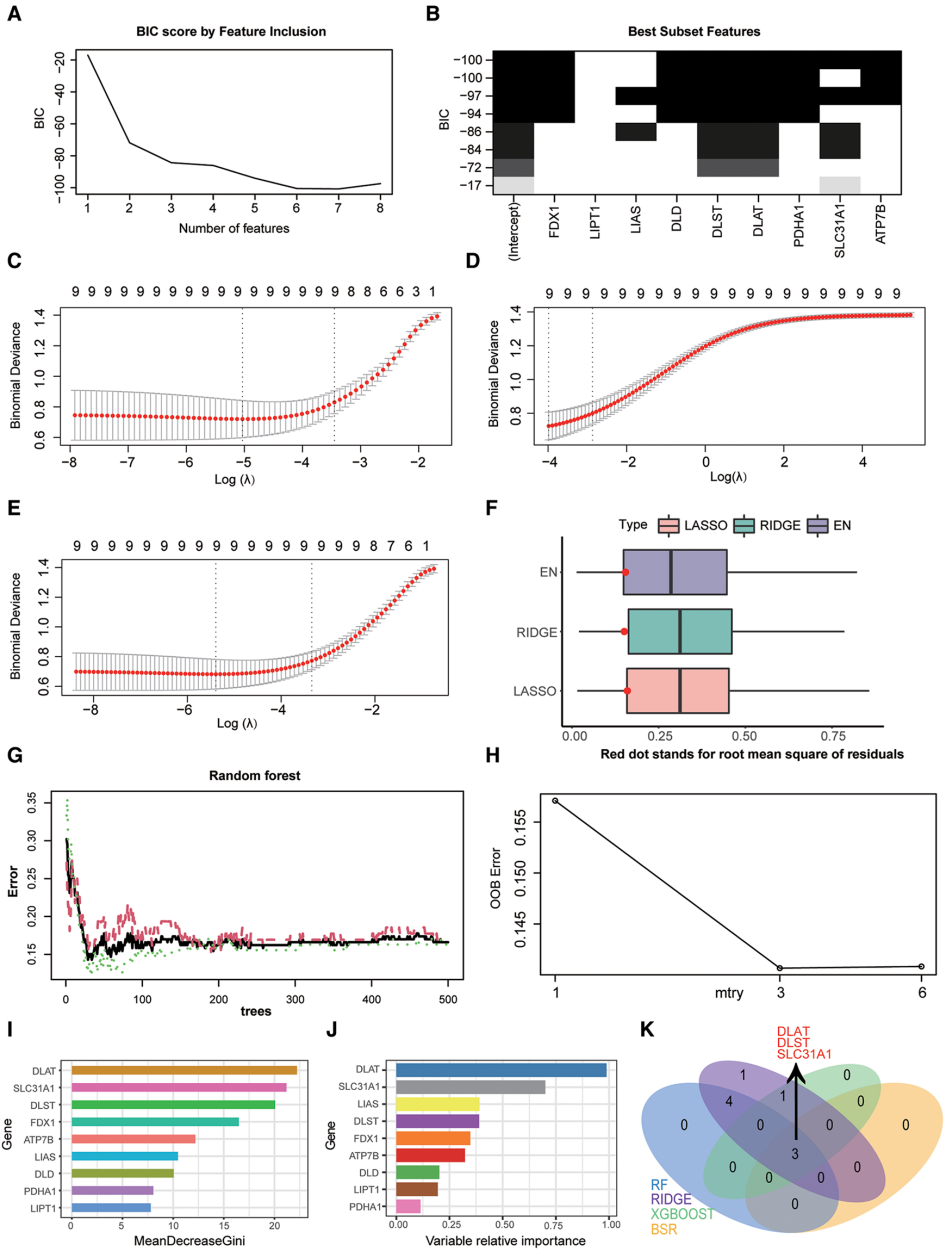
Screening diagnostic markers of CRGs by three feature selection algorithms. (**A**) Bayesian information criterion score by feature inclusion. (**B**) Plot of model performance based on different feature subsets. (**C**) identification of diagnostic markers by LASSO regression algorithm. (**D**) Identification of diagnostic markers by RIDGE regression algorithm. (**E**) Identification of diagnostic markers by EN regression algorithm. (**F**) RMSE of three regularization technique models in the internal validation dataset. (**G**) The influence of the number of decision trees on the OOB error rate. The *x*-axis represents the number of decision trees, and the *y*-axis indicates the OOB error rate. (**H**) Search for the optimal value (with respect to OOB error estimate) of mtry for RF model. (**I**) Results of the Gini coefficient method in RF classifier. The features are ranked by the mean decrease in classification accuracy when they are permuted. The more the Gini coefficient decreases on average, the more important the variable is. (**J**) Results of the supervised integrated learning algorithm of XGBoost. (**K**) Venn diagram showing the intersected genes of four feature selection algorithms. CRGs, cuprotosis-related genes; LASSO, least absolute shrinkage and selection operator; EN, elastic net; RMSE, root mean squared error; OOB, out-of-bag; RF, random forest; XGBoost, eXtreme gradient boosting.

In our study, four feature selection method were applied to determine diagnostic markers of CRGs in HF. The result of best subset regression indicated the BIC was lowest (BIC = −100.73603) for the model with seven CRGs (FDX1, DLD, DLST, DLAT, PDHA1, SLC31A1, and ATP7B) (Figures 4A–B). As for three regularised linear methods, the coefficient profile plot of LASSO, RIDGE and EN was shown in Figures 4C–E. As shown in Figure 4F, RIDGE is the optimum model which produced the minimum RMSE in the internal validation dataset. Moreover, significant features of CRGs were identified by a random forest model, which displayed an accuracy rate of 85.9% with 29 trees and 3 mtry (Figures 4G–H). The contribution of a CRG to the random forest model was evaluated with the mean GINI index decrease. Gini decrease value indicated that DLAT, DLST, and SLC31A1 are important features for the risk evaluation of HF (Figure 4I). Besides, by the supervised integrated learning algorithm of XGBoost, the 4 top-ranked CRGs (DLAT, SLC31A1, LIAS, and DLST) were selected for further analysis (Figure 4J). Taking the intersection of CRGs from best subset regression, ridge regression, RF and XGBoost algorithm, DLAT, DLST, and SLC31A1 were diagnostic markers of CRGs in HF (Figure 4K).

### Development and verification of a CRGs diagnostic signature for HF diagnosis

**Figure 5:**
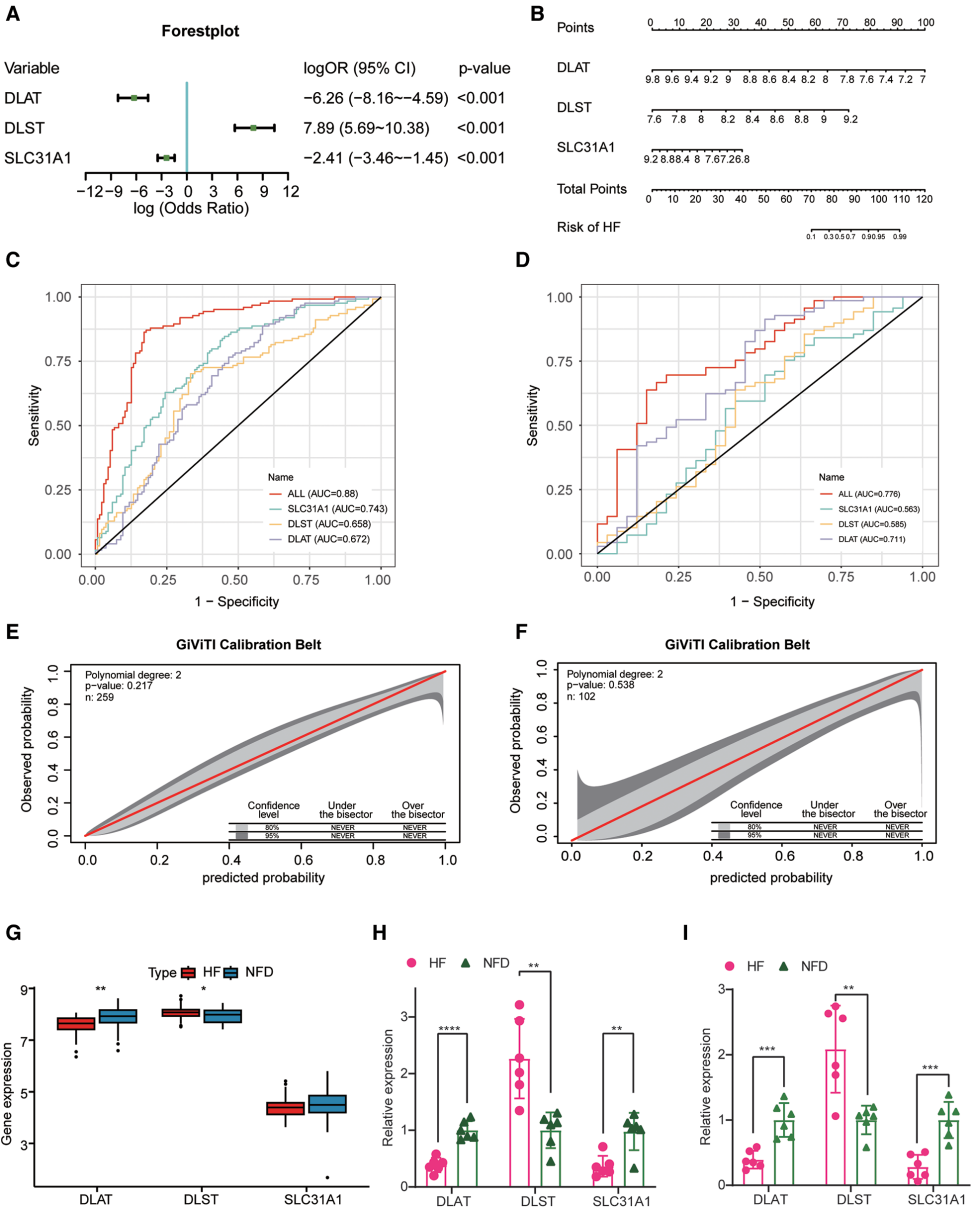
Construction and validation of the CRGs-based diagnostic model. (**A**) A forest plot of the predictive values of three diagnostic markers of CRGs in a multivariate logistic regression analysis. (**B**) Nomogram for predicting probability of HF. (**C,D**) The discrimination capacity of the CRGs signature in the merged dataset (**C**) and external validation dataset (**D**). (**E,F**) The calibration ability of the CRGs signature in the merged dataset (**E**) and external validation dataset (**F**). (**G**) The expression profiles of three CRGs between HF patients and NFDs in the external validation dataset. (**H**) The expression profiles of three CRGs in human heart tissues from HF patients and NFDs. (**I**) The expression profiles of three CRGs in human plasma samples from HF patients and NFDs. ns = not significant, *p < 0.05, **p < 0.01, and ***p < 0.001 vs. the NFD group. CRGs, cuprotosis-related genes; HF, heart failure; NFDs, nonfailing donors.

After identifying three diagnostic markers of CRGs in HF, we used logistic regression analysis to estimate the association between the expression of these diagnostic markers and HF. multivariate logistic regression analysis demonstrated that these three CRGs were independently associated with HF (Figure 5A), and a nomogram was constructed on the basis of the multivariate logistic regression (Figure 5B). In the merged dataset, the nomogram yielded an AUC of 0.880 (95% CI, 0.837–0.922), while in the external validation dataset, the AUC value of this prediction nomogram was 0.776 (95% CI, 0.678–0.874) (Figures 5C,D). The results revealed that this diagnostic signature possessed an excellent prediction performance in classifying HF patients and NFDs, indicating CRGs indeed play a crucial role in HF development. Additionally, the 95% CI region of GiViTI calibration belt did not cross the 45-degree diagonal bisector line in the merged dataset and external validation dataset (*p* = 0.217 and *p* = 0.538; respectively) (Figures 5E,F), implying good consistency between the nomogram-predicted probability of HF and the actual HF status in both datasets. Meanwhile, differential expression of DLAT and DLST were observed in the external validation dataset, further demonstrating their promising diagnostic efficiency (Figure 5G). In addition to external validation of RNA-seq datasets, we performed qRT-PCR experiments to further validate the expression of cuprotosis-related biomarkers using heart tissues and plasma samples from HF patients and NFDs. As shown in Figures 5H,I, DLAT and SLC31A1 were significantly downregulated in the heart tissues or plasma samples of HF patients compared with NFDs (*p* < 0.05), while DLST was significantly upregulated. This finding was consistent with the results of the prior bioinformatics analysis. Overall, the three cuprotosis-related biomarkers demonstrated excellent diagnostic performance for HF. In addition, Supplementary Figure S3 shows DLAT and DLST expression primarily in human ventricular cardiomyocyte, while SLC31A1 expression primarily in atrial cardiomyocyte.

### Correction analysis of diagnostic markers and immune infiltration

**Figure 6:**
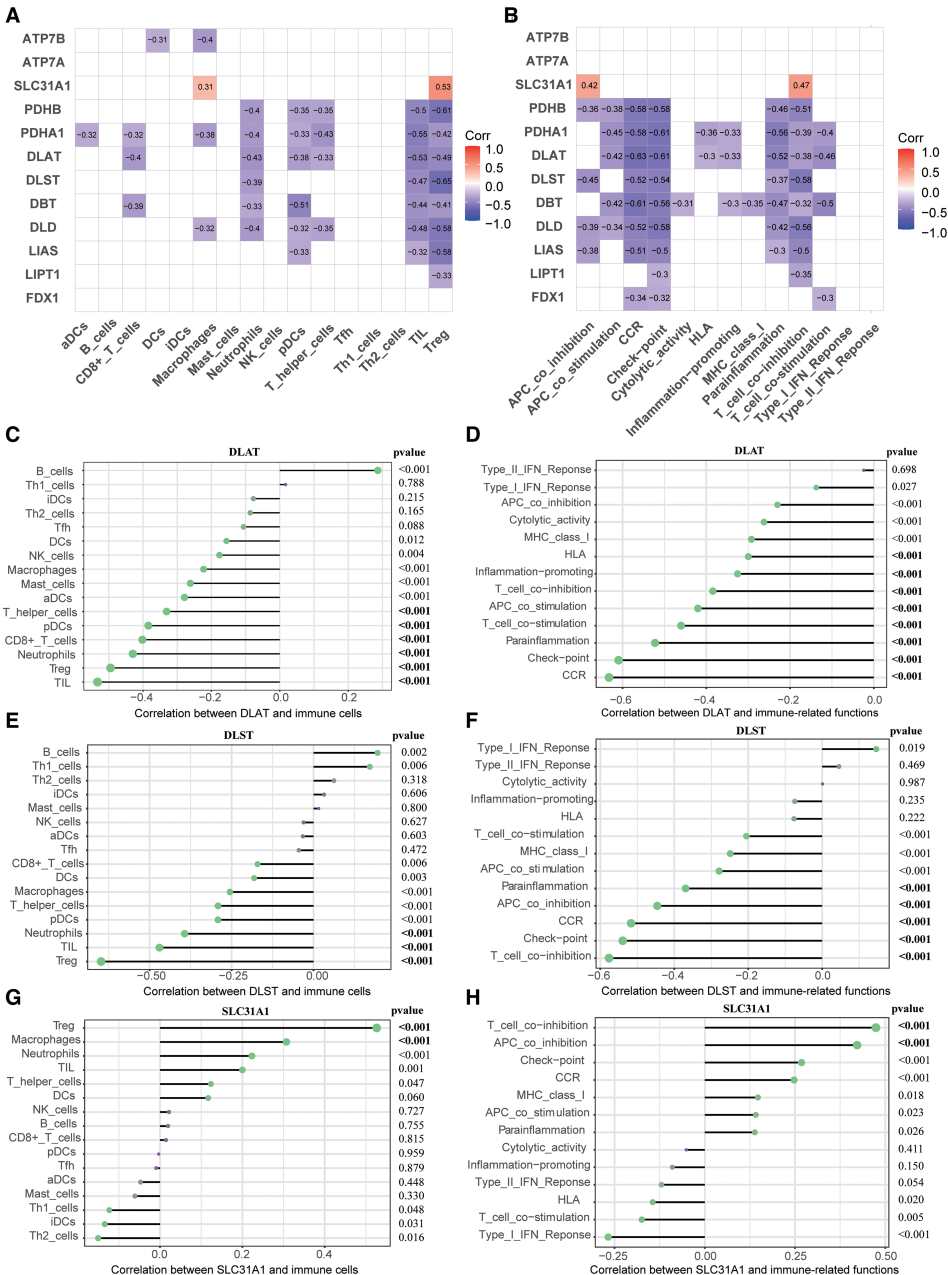
Correlation between CRGs expression and immune characteristics in HF. (**A**) Heatmap of the correlations between 12 CRGs and 16 immune cells. (**B**) Heatmap of the correlations between 12 CRGs and 13 immune-related functions. (**C–E**) Correction between DLAT (**C**), DLST (**D**), SLC31A1 (**E**) and immune cells. (**F–H**) Correction between DLAT (**F**), DLST (**G**), SLC31A1 (**H**) and immune-related functions. CRGs, cuprotosis-related genes; HF, heart failure.

To elucidate association between CRGs and immune infiltration, the correlations between CRGs and 29 immune signatures were explored. We can see that many immune signatures, such as TIL, Treg, CCR, Check-point, Parainflammation, and T cell co inhibition were associated with multiple CRGs (Figures 6A,B). This correlation result indicated that immune dysregulation in HF may be affected by CRGs. Furthermore, we focused on the relationship between three diagnostic markers of CRGs (DLAT, DLST, and SLC31A1) and immune signatures. For immune cells, DLAT was negatively correlated with TIL (*r* = −0.53), Treg (*r* = −0.49), Neutrophils (*r* = −0.43), CD8+ T cells (*r* = −0.40), pDCs (*r* = −0.38), as well as T helper cells (*r* = −0.33); DLST was negatively correlated with Treg (*r* = −0.65), TIL (*r* = −0.47), and Neutrophils (*r* = −0.39); SLC31A1 was positively correlated with Treg (*r* = 0.53) and Macrophages (*r* = 0.31) (Figures 6C–E). In terms of immune-related functions, DLAT showed negative correlation with CCR (*r* = −0.63), Check-point (*r* = −0.61), Parainflammation (*r* = −0.52), T cell co stimulation (*r* = −0.46), APC co stimulation (*r* = −0.42), T cell co-inhibition (*r* = −0.38), Inflammation-promoting (*r* = −0.33), and HLA (*r* = −0.30); DLST showed negative correlation with T cell co inhibition (*r* = −0.58), Check-point (*r* = −0.54), CCR (*r* = −0.52), APC co inhibition (*r* = −0.45), and Parainflammation (*r* = −0.37); SLC31A1 showed positive correlation with T cell co inhibition (*r* = 0.47) and APC co inhibition (*r* = 0.42) (Figures 6F–H).

### Enrichment analysis revealing the potential biological functions of diagnostic markers of CRGs in HF

**Figure 7:**
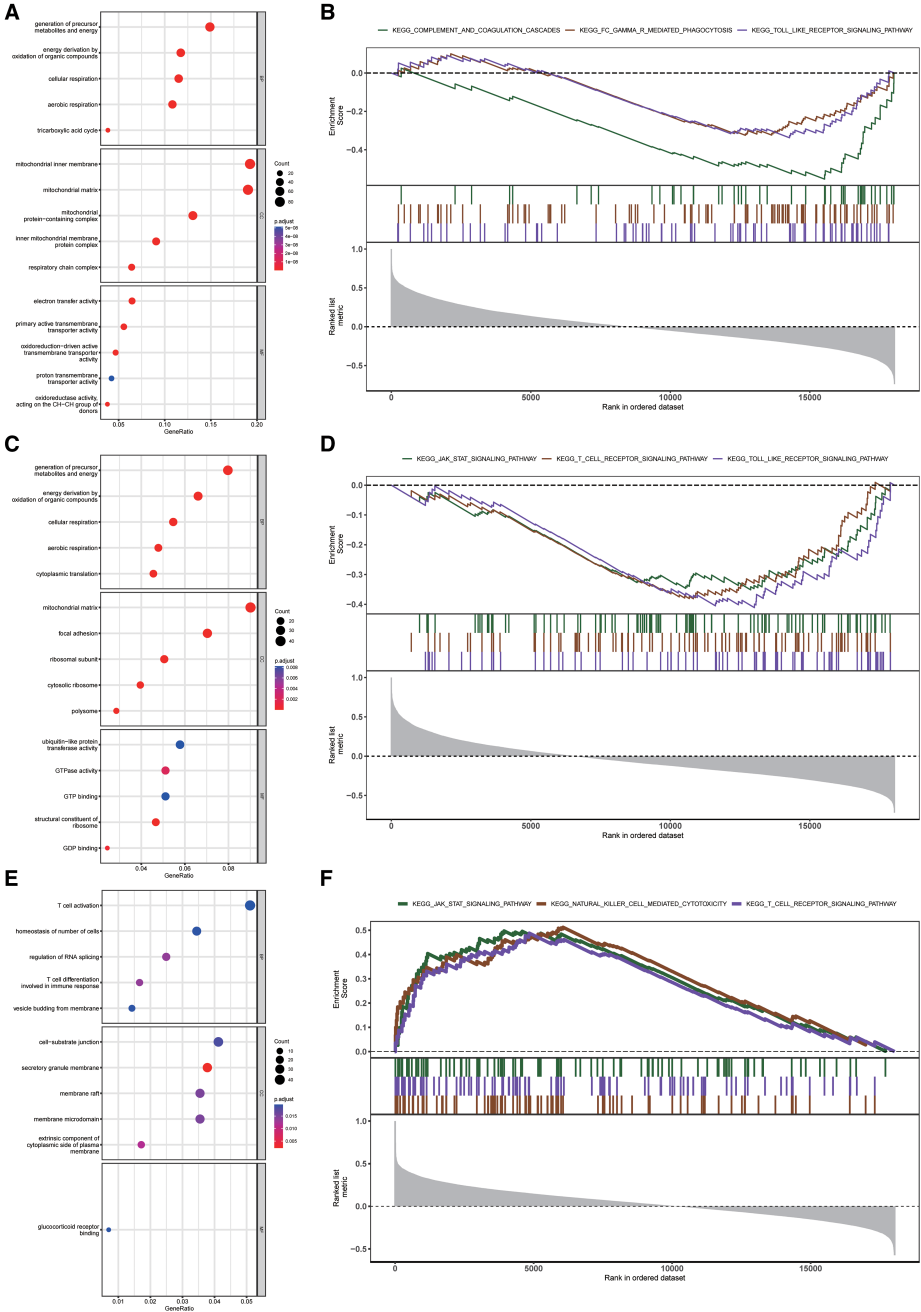
Enrichment analysis of DLAT, DLST, and SLC31A1 in the external validation dataset. (**A–C**) GO function enrichment analysis of DLAT-related genes (**A**), DLST-related genes (**B**), and SLC31A1-related genes (**C**) (**D–F**) KEGG pathway enrichment analysis of DLAT-related genes (**D**), DLST-related genes (**E**), and SLC31A1-related genes (**F**) GO, gene ontology; KEGG, kyoto encyclopedia of genes and genomes.

To reveal the underlying mechanism of three diagnostic markers of CRGs (DLAT, SLC31A1, and DLST) involved in HF, GO and KEGG enrichment analysis was conducted on these CRGs-related genes. DLAT-related genes and DLST-related genes were preferentially involved in biological process related to energy metabolism, while SLC31A1-related genes were significantly enriched in immune-related biological processes (Figures 7A–C). Additionally, GSEA enrichment plot showed that the pathways involved in three CRGs-related genes were immune-related pathways (Figures 7D–F). Together these results demonstrated an important role for these three diagnostic markers of CRGs participating in the regulation of energy metabolism and immune response in HF.

## Discussion

Copper, one of the most abundant transition metals, is essential for survival in the human body (34). It plays a crucial role in many fundamental physiological processes in organisms, including energy metabolism and cellular respiration, collagen and neurotransmitter synthesis, maintenance of blood vessel integrity, and functioning as a redox enzyme involved in the redox regulation (35–37). Precise homeostatic control of copper is vital to the body, and current studies suggest that both excessive copper levels and copper deficiency may lead to pathological conditions including inflammation, neurodegeneration, and cancer (38–40). A recent study conducted by Todd Golub's team provides a novel perspective on the key role of copper in cellular activities, which is termed as “cuprotosis” (13). Cuprotosis is dependent on mitochondrial stress and is induced by direct binding of copper to lipoylated components of the TCA cycle. As a unique form of cell death, cuprotosis is expected to shed light on various diseases, including HF. In our research, we investigated the expression profiles of 13 CRGs in heart tissues of HF patients and NFDs. By employing machine learning methods, we successfully constructed a CRGs diagnostic signature that exhibited powerful predictive capabilities in HF. Furthermore, results from enrichment analyses and immune infiltration showed that the three diagnostic markers of CRGs (DLAT, SLC31A1, and DLST) were associated with energy metabolism and immune activity. This suggests that cuprotosis may be involved in the onset and development of HF through pathways related to energy metabolism and immune regulation.

HF is a chronic disease associated with high mortality and poor prognosis. The pathogenesis of HF is multifactorial and caused by complex mechanisms. Previous studies have found that microelements play a crucial role in the development and progression of HF (41–43). Peculiarly, copper is strongly implicated in the pathological process of cardiac hypertrophy. Copper has the ability to scavenge reactive oxygen species (ROS) and protect cardiomyocytes from ROS-induced damage by binding to zinc (44). Zheng et al. summarized the mechanism of copper supplementation-induced regression of cardiac hypertrophy. These mechanisms include the recovery of cytochrome c oxidase activity and other critical cellular events, the activation of the hypoxia-inducible factor 1 transcriptional complex to inhibit myocardial remodeling through oxygen metabolism pathways, the activation of vascular endothelial growth factor receptor-1-dependent regression signaling pathway in cardiomyocytes, and the inhibition of vascular endothelial growth factor receptor-2 through post-translational regulation in the hypertrophic cardiomyocytes (45). This finding indicated that copper supplementation could be a feasible approach for clinical intervention in HF.

In this study, we used integrated bioinformatics analysis and four feature selection methods to identify cuprotosis-related diagnostic biomarkers in HF. As an active and fruitful research field in machine learning, feature selection algorithm can select the most significant features from the feature space. This not only reduces the classification errors but also shrink the feature space (46). The innovative combination of feature selection approaches highlighted the novelty of our research and improved the predictive ability of our diagnostic model of CRGs in HF. The first method we employed, best subset regression, produces a series of models with an increasing number of predictors. It aims to find out the best-fit model among all possible subsets (47). The second method, regularised regression, is designed to mitigate model overfitting by shrinking coefficient estimates towards zero (48). Third, as an ensemble algorithm, RF has proven to be highly accurate in disease diagnosis and risk prediction (49). Lastly, XGBoost is an integrated machine-learning algorithm based on a decision tree, which is suitable for classification, regression, sorting, and other problems. Utilizing these four feature selection methods, we identified three CRGs (DLAT, DLST, and SLC31A1) as potential diagnostic markers in HF. Internally and externally validated results demonstrated that the model featuring three CRGs exhibited good discrimination and calibration for predicting HF. DLAT encodes component E2 of the multi-enzyme pyruvate dehydrogenase complex (PDC), responsible for the oxidative decarboxylation of pyruvate, producing acetyl-CoA and CO2. The phytochemical hyperforin can trigger thermogenesis in adipose tissue and increase energy expenditure via a DLAT-AMPK signaling pathway, making it a promising approach for obesity therapy (50). DLST is a key component of the *α*-ketoglutarate dehydrogenase complex which participates in the process of oxidative decarboxylation in TCA cycle. Inhibition of microRNA-146a and overexpression of its target DLST have been shown to alleviate pressure overload-induced cardiac hypertrophy and dysfunction (51). SLC31A1 acts as a high affinity copper uptake transporter, which is responsible for facilitating the uptake of approximately 80% of copper into cells (52). Angiogenesis is a complex process regulated by multiple factors, among which VEGF plays a vital role in crucial process (53). Oxidation of SLC31A1 at its cytosolic Cys189 residue can enhance VEGFR2 internalization and signaling, consequently promoting angiogenesis (54). These results suggest that these cuprotosis-related biomarkers may be involved in the development of HF through regulating in aerobic respiration of cardiomyocytes.

Immune-mediated mechanisms are believed to play a critical role in the pathogenesis of HF. Multiple studies have demonstrated the detrimental effects of immune cells in myocardial remodeling, but also their potential role as essential mediators of cardiac repair (55–57). In order to further explore the role of immune infiltration in HF, we utilized ssGSEA to evaluate the correlations between CRGs and immune characteristics of HF. Our findings revealed meaningful correlations between Treg cells and multiple CRGs. Specifically, it showed that Treg cells had a negative correlation with DLAT and DLST, as well as a positive correlation with SLC31A1. Treg cells have the ability to suppress a variety of immune responses, thus contributing to immune homeostasis (58). Through exerting proinflammatory and antiangiogenic effects, Treg cells can promote immune activation and pathological left ventricular remodeling in the progression of chronic ischemic HF. Restoring normal Treg cell function may therefore represent a promising target for therapeutic immunomodulation in HF (59). Additionally, T cell co-inhibition was significantly associated with three cuprotosis-related biomarkers. T cell response is modulated by inflammatory signals and contribute to the onset and progression of cardiovascular disease. Previous study has highlighted the importance of regulatory mechanisms of T cell co-inhibition pathways for controlling the T cell response and treating cardiovascular disease (60). In addition, KEGG pathway analyses revealed notable enrichment of immune response-related pathways in relation to the three cuprotosis-related biomarkers in HF. The role of inflammation and immune dysfunction in HF is widely recognized. Immune cells, particularly T cells, are influenced by inflammatory signals and contribute to the development and progression of HF. Based on our correlation and enrichment findings, we speculate that the three cuprotosis-related biomarkers may participate in the occurrence and progress of HF through modulation of T cell-related pathways.

Yet our study has presented some deficiencies. Firstly, limited by accessing human heart tissues, although as many qualified GEO database of HF samples as possible were included in our study, the sample size was still small. More HF datasets are needed to validate our diagnostic model of CRGs and improve it. Especially, human heart single-cell sequencing datasets are needed to verify the expression of CRGs in different populations of cells in the heart. Secondly, the lack of detailed clinical information in the GEO datasets prevents the analysis of the correlation between cuprotosis-related biomarkers and the clinical characteristics of HF patients. Thirdly, the subject of our study is human heart rather than serum sample. It is necessary to explore whether these indicators of cuprotosis in serum can be used as diagnostic biomarkers for HF. Fourthly, there are many causes of HF, including hypertension, ischemic cardiomyopathy, dilated cardiomyopathy, valvular and congenital heart disease, arrhythmias and degenerative cardiomyopathies. Considering the heterogenous aetiology of HF, further molecular biology experiments are required to investigate the function and regulation mechanism of cuprotosis biomarkers in different aetiologies of HF. Additionally, it is important to note that the conclusions drawn from our paper are primarily based on bioinformatics analysis. Consequently, further experiments are necessary to gain a deeper understanding of the mechanisms underlying cuprotosis and the role of CRGs in the progression of HF.

## Conclusions

In conclusion, this study is the first comprehensive exploration into the role of cuprotosis regulators in HF. Using the combination of four machine learning algorithms, we developed a diagnostic model incorporating three CRGs, which exhibited excellent diagnostic performance in HF. Besides, the results of immune infiltration and enrichment analysis further revealed that cuprotosis and CRGs is associated with multiple immune signatures and pathways. Therefore, it may be used to develop a novel strategy for the immunotherapy of HF.

## Data Availability

The datasets presented in this study can be found in online repositories. The names of the repository/repositories and accession number(s) can be found below: https://www.ncbi.nlm.nih.gov/, GSE16499, GSE26887, GSE42955, GSE57338, GSE76701, and GSE79962.
