# Supplementary material for: Integrative analysis of bioinformatics and machine learning to identify cuprotosis-related biomarkers and immunological characteristics in heart failure
Source: Front Cardiovasc Med. 2024 Mar 18;11:1349363. doi: 10.3389/fcvm.2024.1349363 (PMC10982316; doi:10.3389/fcvm.2024.1349363)
Supplement: Supplementary file 3 [file Datasheet2.pdf]

Supplementary Material for

Integrative analysis of bioinformatics and machine learning to identify cuprotosis-related biomarkers and immunological characteristics in heart failure

Table S1. The characteristics of six screened microarray datasets of HF in GEO database
Table S2. The characteristics of one validation RNA-seq dataset of HF in GEO database
Table S3. Summary of 13 cuprotosis-related genes
Figure S1. Principal component analysis plots of batch-removed expression data of six HF microarray datasets
Figure S2. Normalizing gene expression matrices of the merged external validation dataset

Abbreviations: RNA-seq, RNA-sequencing; HF, heart failure; ICM, ischemic cardiomyopathy; DCM, dilated cardiomyopathy; NFD, nonfailing donor; GEO, Gene Expression Omnibus; PCA, Principal Component Analysis.

Table S1. The characteristics of six screened microarray datasets of HF in GEO database

Dataset ID	Country	Number of samples			Microarray platform
		HF		NFD	
		ICM	DCM		
GSE16499	USA	15	0	15	Affymetrix Human Exon 1.0 ST Array
GSE26887	Italy	7	0	5	Affymetrix Human Gene 1.0 ST Array
GSE42955	Spain	0	24	5	Affymetrix Human Gene 1.0 ST Array
GSE57338	USA	27	27	95	Affymetrix Human Gene 1.1 ST Array
GSE76701	USA	4	0	4	Affymetrix Human Genome U133 Plus 2.0 Array
GSE79962	USA	11	9	11	Affymetrix Human Gene 1.0 ST Array

Table S2. The characteristics of three validation RNA-seq datasets of HF in GEO database

Dataset ID	Country	Number of samples			Platform ID
		HF		NFD	
		ICM	DCM		
GSE116250	USA	13	37	14	GPL16791
GSE71613	Italy	0	4	4	GPL11154
GSE48166	USA	15	0	15	GPL9115/GPL9442

Table S3. Summary of 13 cuprotosis-related genes

Gene	Type
FDX1	cuprotosis-related genes
LIPT1	cuprotosis-related genes
LIAS	cuprotosis-related genes
DLD	cuprotosis-related genes
DBT	cuprotosis-related genes
GCSH	cuprotosis-related genes
DLST	cuprotosis-related genes
DLAT	cuprotosis-related genes
PDHA1	cuprotosis-related genes
PDHB	cuprotosis-related genes
SLC31A1	cuprotosis-related genes
ATP7A	cuprotosis-related genes
ATP7B	cuprotosis-related genes

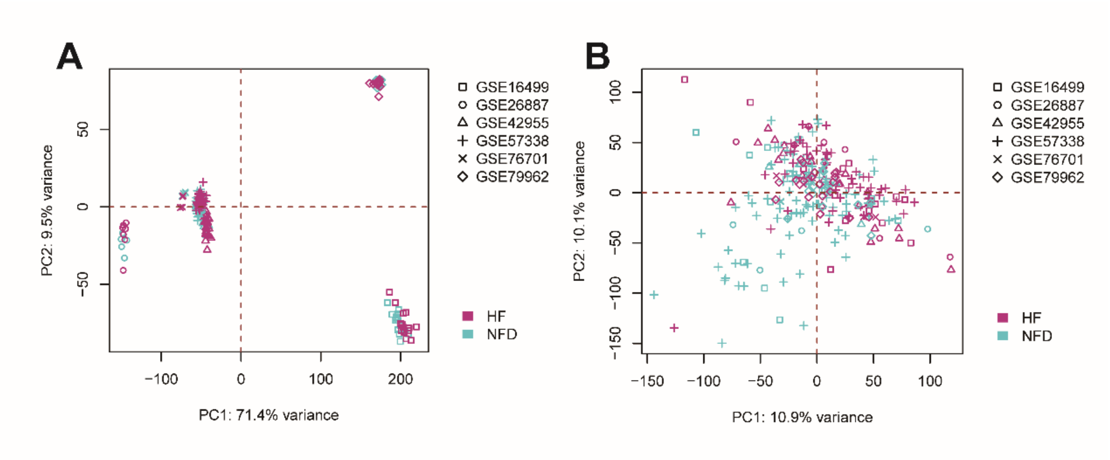

Figure S1. PCA plots of batch-removed expression data of six HF microarray datasets. The batch effect was removed by using the "Combat" algorithm in the sva R package. **A.** Before batch effect removal; **B.** After batch effect removal.

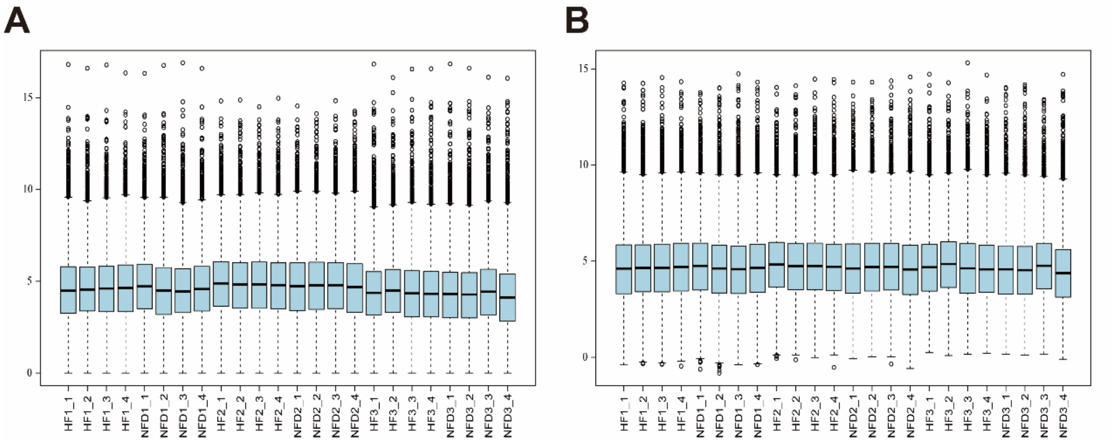

Figure S2. Normalizing gene expression matrices of the merged external validation dataset. The batch effect was removed by using the R package "RUVSeq". For each validation dataset, eight samples (four HF, four NFDs) were selected to demonstrate the efficacy of batch effect removal. **A.** Before batch effect removal; **B.** After batch effect removal

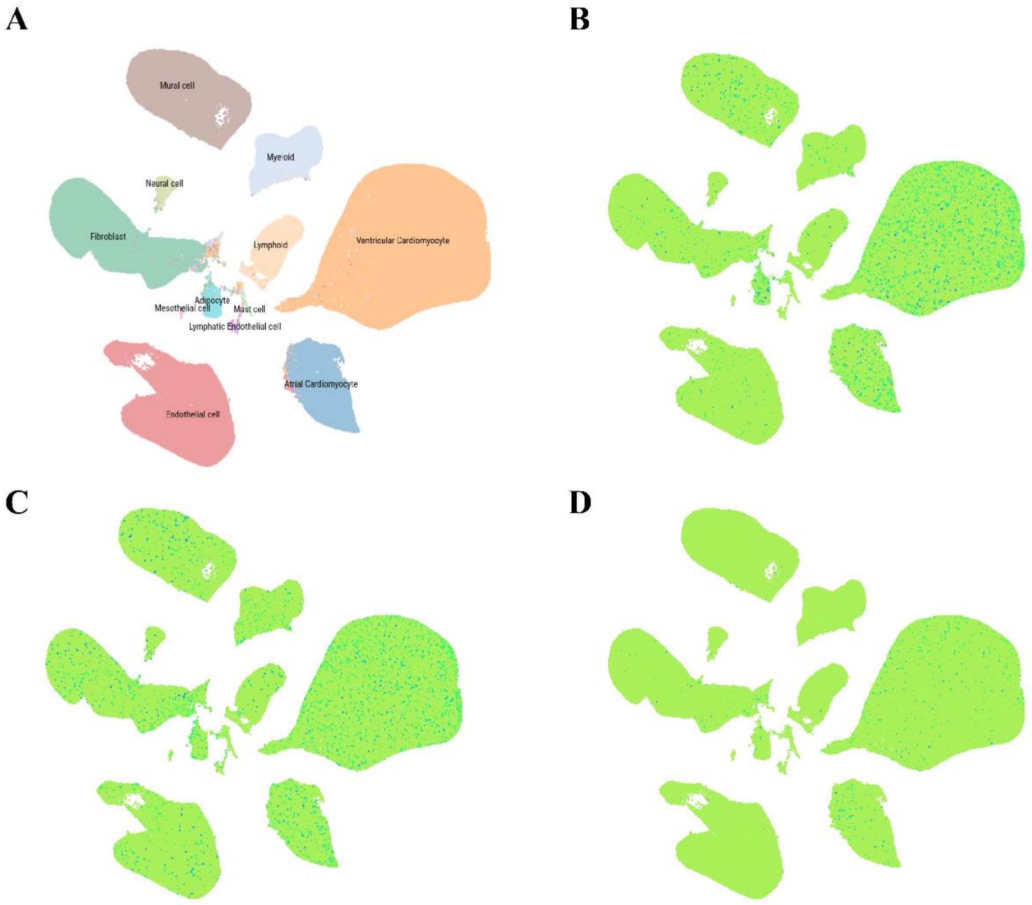

Figure S3. Human cardiac DLAT, DLST and SLC31A1 expression. **A.** Uniform manifold approximation and projection for dimension reduction (UMAP) visualization of human cardiac cells. **B.** DLAT expression in human cardiac cell population. **C.** DLST expression in human cardiac cell population. **D.** SLC31A1 expression in human cardiac cell population.
